# Teledermatology Evaluation and Feedback Systems: A Tool for Improving Care

**DOI:** 10.3390/healthcare11111626

**Published:** 2023-06-02

**Authors:** José Luis Gatica, Diego Aragón-Caqueo, Gabriel Aedo, Héctor Fuenzalida, Rodrigo Loubies, Sócrates Aedo, María Francisca Carrasco, Vezna Sabando, Carolina Cunill, María José Letelier

**Affiliations:** 1 Hospital Digital, Célula de Teledermatología, Ministerio de Salud de Chile, Santiago 8320218, Chile; joseluisgaticamonsalve@gmail.com (J.L.G.); mariafrancisca.carrasco@minsal.cl (M.F.C.); vezna.sabando@minsal.cl (V.S.); carolina.cunill@minsal.cl (C.C.); mjletelier@minsal.cl (M.J.L.); 2 Escuela de Medicina, Universidad de Tarapacá, Arica 1000000, Chile; 3 Facultad de Ciencias Médicas, Universidad de Santiago de Chile, Santiago 8320000, Chile; gabrielaedoinostroza@gmail.com (G.A.); hector.fuenzalida@usach.cl (H.F.); rloubies@gmail.com (R.L.); 4 Escuela de Medicina, Facultad de Medicina, Universidad Finis Terrae, Santiago 7501015, Chile; socrates.aedo@gmail.com

**Keywords:** teledermatology, information systems, quality of care, feedback systems

## Abstract

Chile is a country where teledermatology has been growing exponentially since the implementation of a single national asynchronous teledermatology platform for the public system in December 2018. To ensure the quality of care in teledermatology systems, it is crucial to evaluate the fulfillment of basic specifiers such as ICD-Diagnosis, therapeutic suggestions, and diagnostic suggestions, among others. This article aims to evaluate the teledermatology system of the Chilean public health service by analyzing 243 randomly extracted consultations, representative of the 20,716 electronic consultations performed during 2020. Compliance with basic specifiers is evaluated. From these, fulfillment of core teledermatology functions, such as diagnostic and therapeutic suggestions, is observed in most consultations. There are statistically significant relationships between the patient’s destination (primary health center [PHC] or face-to-face referral), pharmacological prescription, coverage of the drug prescribed by the public system, and the education received by the consulting physician. If the consultation is resolved in the PHC, there is a higher chance for pharmacological prescription, prescribing mostly drugs that are covered by the government. This is less likely to occur when patients are referred for face-to-face evaluation. A targeted evaluation of education, pharmacological prescriptions, and their applicability is key to improving the quality of teledermatology systems.

## 1. Introduction

The advance in information and communication technologies has promoted tools such as telemedicine as a viable alternative for delivering health care [1,2]. Teledermatology (TD) is a branch of telemedicine that has gained a presence in the healthcare systems over the last decade [3]. Due to the visual nature of the skin examination and the visual expression of dermatological diseases, the use of TD rises as a potential tool for diagnosing and managing skin conditions by distance [4]. Given that the diagnostic workup in dermatology mostly relies on visual clues, the clinical presentation of most dermatological conditions can be documented by imagining technologies, making the specialty ideally suited for this model of delivering specialized healthcare attention and supporting clinical decision-making [3].

Studies regarding the implementation of TD systems within the healthcare systems, as a tool to support access to specialized care, have shown comparable outcomes to conventional face-to-face consultation in terms of accuracy in diagnosis, management, and clinical outcomes, in settings where face-to-face consultation is not available or access to specialist consultation is severely limited [5,6,7]. In addition, several studies suggest that TD is a cost-effective tool that allows for the reduction of waiting times and efficient use of specialized resources and optimizes the relevance of referrals to face-to-face evaluation by the specialty, ultimately improving access to health care in populations with limited access [8,9,10,11].

Modern-day TD is usually divided into three different modalities: live interactive, store-and-forward, and a hybrid model including both of the previously mentioned modalities. The most used and studied modality is the store-and-forward TD model, where a clinician, usually at a primary healthcare center, summarizes the clinical history and relevant findings of the physical examination of a patient, captures images of the lesion or lesions involved, and uploads them into a TD platform. Then, a dermatologist evaluates the consultation and delivers a diagnostic and therapeutic suggestion. When compared to the live interactive model, it has lower equipment costs and improved utilization of specialist resources, and patients feel more comfortable [12]. Nevertheless, the use of live interactive or hybrid models can be of greater value for certain clinical scenarios such as case follow-up or to support clinical decision-making when the suspected pathology requires timely intervention.

For the particular case of Chile, given the geographical and demographical distribution of the country, developing a TD platform for the public system to reach isolated communities is considered a public health measure of utmost importance [13]. Note that Chile is a long and vast territory with 4329 km in length, poor land connectivity between cities, with a rurality index of 12.1% and 15% of the territory corresponding to islands [14]. Furthermore, there is an uneven distribution of specialists across the country, where one-third of the dermatologist’s available work for the public sector, and from them, more than a half concentrate in the central regions of Chile, specifically in Santiago, the capital of Chile, and its surroundings [15]. This uneven distribution leads to an over-representation of specialists based on the population they cover in urban centers, while more isolated areas have little to no access to specialized care [16,17]. In addition, access to dermatological care in the public health system in Chile has been historically limited, with a documented gap between supply and demand that makes dermatology one of the highest-demanded specialties in the country [18]. Note that the public system covers 80% of the Chilean population, and dermatology consultation accounts for 2.7% of all consultations for specialized care in the public sector [17]. This, in turn, determines a considerable limitation on access to dermatological consultation, which translates into long waiting times, averaging 341 days for face-to-face dermatological evaluation [19].

Given this context, in December 2018, the Chilean Ministry of Health implemented a TD platform for the public sector, covering all primary healthcare centers of the Chilean territory [20]. This project was part of a greater project called Digital Hospital, which aims to implement telemedicine systems across the specialties of the public sector in order to promote timely and efficient access to specialist evaluation, regardless of the geographical location of the patient [20]. For the case of dermatology, the platform works as a store-and-forward TD system, where the primary healthcare physician, with the previous consent of the patient, takes images of the dermatological lesion or lesions involved in the case and uploads them to the TD platform, along with the clinical history and main findings of the physical examination. From there, a dermatologist at an urban center evaluates the case and gives diagnostic and therapeutic suggestions. In addition, to strengthen primary care, a brief education from the specialist regarding the suspected pathology is usually included in the response.

In the Chilean healthcare system, the primary health centers function as a gateway to the secondary and tertiary levels of attention, where most of the specialized resources are found. The general practitioner of the primary care is in charge of solving common pathology and referral to the specialist if needed, under established criteria, based on the diverse healthcare programs that organize the flow of referral to secondary and tertiary levels on each specialty. For the case of dermatology, this flow of patients to more specialized levels of attention should start with the case evaluation via the TD platform, where a specialist can suggest referral to face-to-face evaluation with a dermatologist or can suggest management in the primary healthcare center. Nevertheless, if the primary healthcare physician suspects a specific dermatological disease that has to be managed by specialists, direct referral to specialty centers is always available.

Originally, the TD system was first envisaged as a support tool for the general physician in primary care with rapid specialist suggestions on the case. However, it is now being implemented as a common channel for referral to dermatology and not limited to cases where the primary care physician is uncertain of the diagnosis or management. Nowadays, Chile is experiencing a transitional phase in the referral system to the specialty, introducing the TD platform as the initial step for specialist evaluation.

Regarding pharmacological interventions, the public system delivers pharmacological coverage for most first-line treatments of diverse dermatological pathology. This is achieved either by the coverage offered by the primary healthcare, the coverage offered by the telemedicine program, or the coverage offered by the secondary level. In the case of primary healthcare, primary healthcare centers have a set of established topical and systemic drugs used to treat common skin pathology. These are prescribed by the primary healthcare physician and are covered by the public system, at no out-of-pocket expenditure for the patient. Note that the availability and diversity of this pharmacological arsenal vary according to each specific primary healthcare center, depending on the resources and local supply of drugs.

Moreover, the telemedicine program in the TD cell includes coverage of first-line treatment associated with specific diagnoses, offering treatment coverage for acne, rosacea, psoriasis, non-specified dermatitis, vitiligo, and fungal infections. These drugs are indicated by the dermatologist in the therapeutic suggestion included in the response to the teleconsultation. Afterward, treatment is prescribed in the primary healthcare setting, with adequate supervision from the specialist. Finally, if the patient is referred to the secondary level for specialist evaluation, the secondary level also delivers coverage of drugs that are commonly used in dermatology.

Recent studies on the implementation of the Chilean TD program in the public sector show that 57.55% of the consultations continue management in the primary health centers [13], without the need for a face-to-face evaluation by the specialist, highlighting the resolutive role of the primary health and thus optimizing referral to the secondary level. In addition, there is an average response time of 3.5 days [13], which promotes timely and efficient access to diagnostic and therapeutic suggestions by a specialist.

Nevertheless, when it comes to telemedicine systems, to ensure the best possible service, it is crucial to constantly evaluate their quality [21] since failures in this area can lead to substantial diagnostic and therapeutic errors [22]. There are several methods to evaluate the quality of telemedicine systems, most adopting a qualitative approach [23]. Thus, the evaluation of TD systems implemented throughout different healthcare systems is based mostly on indicators such as user satisfaction [24,25], physician satisfaction [26], education systems [27], and economic impact [28]. However, there are no current publications on audited data on asynchronous electronic consultations to assess the quality of information processing, general practitioner education, organization, and information storage.

In this sense, the current standards of the British Association of Dermatologists (BAD) recommend auditing TD systems data at least once a year to assess the quality of these systems [29]. This article aims to show the evaluation and feedback system of the TD system from the Chilean public health service, by auditing the data of the electronic consultations received during the year 2020.

Large telemedicine systems often have a considerable loss of information due to errors in digiting the response. From this, targeted evaluation of the compliance with basic TD specifiers such as ICD-10 coded diagnosis, therapeutic suggestion, therapeutic indication, the applicability of the response, education to the consulting physician, and final destination of the patient is analyzed on the response from the specialist.

## 2. Materials and Methods

Cross-sectional, observational study of asynchronous electronic consultations uploaded to the TD platform of the Digital Hospital project, dermatology cell from the Ministry of Health of Chile (MINSAL) in 2020. From the universe of the consultations with responses during 2020, a representative sample size is randomly withdrawn and audited. The representative sample size was calculated according to the guidelines proposed by the Chilean Ministry of Health for sample size calculation in healthcare systems quality evaluation [30].

### 2.1. Patients and Settings

As mentioned in the introduction, the TD platform of the public health system covers all the primary healthcare centers of the Chilean territory.

The patients included in the registry are patients that consult at any given primary healthcare center with a specific skin disease. Patients are evaluated by a general practitioner, who requires a diagnostic or therapeutic suggestion by a specialist, to either treat the patient’s pathology in the primary healthcare setting or referral to the secondary level.

### 2.2. Procedure

All patients who require a specialist evaluation for a given dermatological disease in the primary healthcare setting are evaluated by a general physician, who summarizes the clinical history and main clinical findings of the physical examination and takes clinical images of the lesion or lesions involved, with the patient’s consent. These clinical images include a macroscopic picture of the lesion showing its topographic distribution, a macroscopic picture of a single lesion, a zoom-in capture of a single lesion, and a dermoscopic image if applicable and if a portable dermatoscope is available at the center. Afterward, the clinical images and medical history are uploaded to the TD platform. Then, the case is assigned to a dermatologist working at a referral center according to the availability of telemedicine hours for a given dermatologist. At the time of the study, there were 43 dermatologists working on the TD platform. Once the case is evaluated and the response from the specialist is recorded, targeted evaluation of basic specifiers that ensure the quality and usefulness of the response is analyzed.

From a representative sample size of the 20,716 teleconsultations recorded in 2020 on the TD platform of the public system, the responses are evaluated according to compliance with basic specifiers found in Table 1. If a patient is prescribed a certain drug associated with the diagnoses of acne, rosacea, non-specified dermatitis, vitiligo, psoriasis, and fungal infections, the drugs are available to the patient at no out-of-pocket expenditure. The applicability of the response is evaluated as a pharmacological prescription that is included in the drugs covered by the government, either through primary healthcare or through the telemedicine program. A more detailed description of the basic specifiers evaluated can be found in Table 1.

Compliance greater than or equal to 80% with an error margin of 5% is considered the threshold for compliance (Table 2).

The test hypothesis is evaluated through Pearson’s Chi-square test to assess whether there are statistically significant relationships between the variables of the presence of therapeutic suggestion versus resolution in primary healthcare, resolution in primary healthcare versus drug prescription, drug prescription versus drug covered by the public system, and finally patient’s destination (either resolution in the primary healthcare or referral for face-to-face specialist evaluation) and education received by the physician making the teleconsultation.

In addition, a descriptive analysis of the place of resolution of the consultation, together with the most frequent diagnoses, is included.

### 2.3. Statistical Analysis

The descriptive analysis of the demographic variables and the analysis of pathologies diagnosed were performed using Stata software (Stata/SE 16.0 for macOS, Copyright 1985–2019 StataCorp LLC, College Station, TX, USA). All figures were developed using Microsoft Excel (Office 365, Microsoft Excel v16.66.1 for macOS, Copyright 1985–2022 Microsoft Crop, Redmond, WA, USA). Chi-Square test was used to test for statistically significant relationships between the destination of the patient (primary healthcare or Face-to-face referral), pharmacological prescription (yes or no), the applicability of the prescription (whether the drug indicated is included in the basket covered by the public healthcare system), and education received by the physician who makes the consultation in the primary healthcare (yes or no).

### 2.4. Ethical Aspects

The data analyzed were obtained from an anonymous database from the TD Cell of the Digital Hospital project of the Chilean Ministry of Health. The study was approved by the Ethics Committee of the Servicio de Salud de Antofagasta, Antofagasta, Chile. The analysis was conducted in compliance with the Declaration of Helsinki of ethical principles for medical research.

## 3. Results

From the total universe of 20,716 electronic consultations evaluated during 2020, a representative sample size of 243 consultations was randomly extracted.

**Figure 1:**
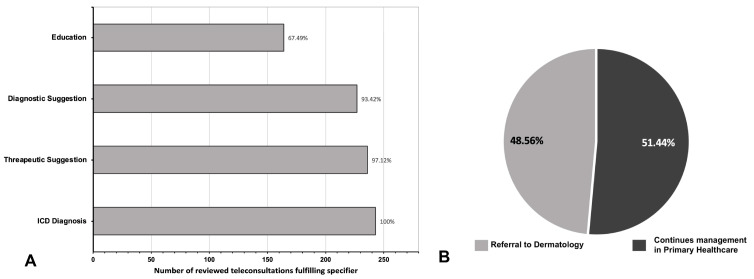
(**A**) Fulfilment of core teledermatology functions as targeted evaluation of specifiers in the response of the reviewed teleconsultations. (**B**) Final destination of patients after the teleconsultation.

The most frequent diagnoses observed were contact dermatitis 8.23% (*n* = 20), psoriasis 5.35% (*n* = 13), viral warts 4.94% (*n* = 12), atopic dermatitis 4.94% (*n* = 12), seborrheic keratosis 4.53% (*n* = 11), and unspecified skin malignancy 3.29% (*n* = 8). On the other hand, the presence of basic specifiers is summarized in Figure 1.

**Figure 2:**
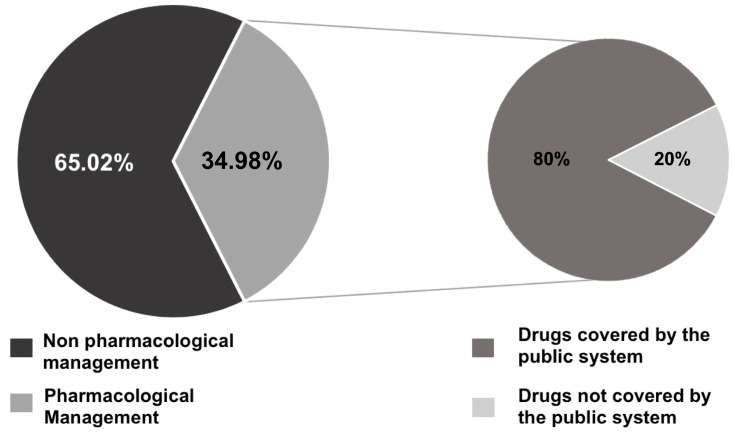
Management indicated in the interconsultation and therapeutic applicability (covered vs. not covered by the public system) when a pharmacological indication was prescribed.

Regarding management, non-pharmacological management was present in 65.02% (*n* = 158) of patients, while pharmacological indication was observed in 34.98% (*n* = 85) of cases. Of the 85 patients that were prescribed a drug, 80% (*n* = 68) were drugs covered by the public system, while 20% (*n* = 12) were drugs not covered by the public system. (Figure 2).

For diagnostic suggestion, 115/125 (92%) patients that continued treatment in primary healthcare received a diagnosis. On the other hand, 112/118 (94.92%) referred to face-to-face evaluation did so. The destination of the patient and the presence of a diagnostic suggestion did not show statistically significant relationships (*p* = 0.84).

Moreover, therapeutic suggestions in patients that continued management in primary care were present in 123/125 (98.4%) consultations. In turn, a therapeutic suggestion in patients referred to face-to-face care was present in 113/118 (95.76%) of these consultations. Pearson’s Chi-square test showed that these variables were independent (*p* = 0.219).

For pharmacological indication, patients that continued management on the primary healthcare were prescribed a drug in 61/125 (48.80%) locations. The pharmacological indication in patients referred to face-to-face care was present in 24/118 (20.34%) cases. There was a statistically significant relationship between the patient’s destination and drug prescription (*p* < 0.01).

Regarding the pharmacological indication itself, 68/85 (80%) of the patients had prescribed drugs covered by the public health system, regardless of their place of destination. In addition, 100% of the patients who continued management in primary healthcare received drugs covered by the public health sector. For those referred to face-to-face evaluation with a pharmacological prescription, 29% of patients received drugs included in the public plan. There is a significant relationship (*p* < 0.001) between the patient’s destination and the pharmacological indication of a drug included in the public health plan.

Finally, for education, there is a statistically significant relationship (*p* = 0.004) between the patient’s destination after consultation and the education received by the primary healthcare physician. From this, 95/125 (76%) of the teleconsultations that resolved in primary care received education from the dermatologist. In contrast, 69/118 (58.47%) of the consultations that were referred to face-to-face evaluation did so.

## 4. Discussion

The evaluation of telemedicine systems is key for improving the quality of care and ensuring the best possible service [31]. For TD systems, the standard recommendation is that data audition and quality evaluation should be performed yearly [29]. In Chile, TD is a field experiencing accelerated growth following the implementation of a single national TD platform for the public system in late 2018 [13]. Since this platform covers all the primary healthcare centers of the Chilean territory, ensuring efficient information processing, storage, and compliance with basic specifiers in the response is of utmost importance. Given this context, this is the first audit of the Chilean TD system since the implementation of the aforementioned platform.

As information processing, clinical records, and information flow in large telemedicine systems are key for the adequate functioning of the platforms and suppose a logistical challenge in expanding telemedicine systems, evaluating the proper functioning of those processes is of utmost importance.

From the specifiers established in Table 1, it is observed that the vast majority of consultations comply with the basic functions that are expected from a TD system. Diagnostic and therapeutic suggestions are found in most of the consultations evaluated (Figure 1A). This implies that core TD functions, such as supporting the primary care physician with diagnostic and therapeutic assistance, are efficiently fulfilled. However, ICD-coded diagnosis was recorded in 100% of the consultations because it is a mandatory field in the Chilean TD platform; thus, 100% compliance is expected. Although ICD diagnosis is recorded, it is also of utmost importance to record the diagnostic suggestion, as the diagnosis given by the specialist might be more specific and helpful and provide greater insight into the case, than the standardized ICD-coded diagnosis. Nevertheless, it is also valid if the professional evaluating the teleconsultation estimates that the diagnosis provided in the ICD code is sufficient to provide the diagnosis, and thus, it does not record a more specific diagnosis suggestion on the response.

Regarding the educational role of TD, education by the dermatologist to the primary care physician is present in 164 (67.49%) of the referrals. For more than three-quarters of the consultations resolved in primary healthcare, the primary healthcare physician received education regarding the pathology from the consulting dermatologist. In contrast, when these consultations were destined for face-to-face evaluation, fewer dermatologists performed an adequate discussion of the case. Considering the important educational role of TD systems, case discussions and constant feedback between the specialist and the general physician help strengthen the role of primary care in solving and referring adequately and thus should be performed in most teleconsultations. This ultimately improves the appropriate use of the platform and optimizes the flow of attention, generating a positive loop between the primary healthcare physician and the dermatologist at the referral centers. This is further supported by a considerable amount of evidence that suggests that telemedicine education has helped medical residency programs [27], general practitioners [32], and patients [33].

In terms of management in the primary healthcare by TD, 51.44% (*n* = 125) of the teleconsultations continued treatment in the institution of origin, with a face-to-face referral of 48.56% (*n* = 121). Similar studies published in Chile show that management of dermatological disease in primary care, with the support of TD, can be as high as 64%, with a face-to-face referral of 30.1% [19]. In turn, articles on TD in Spain show a 50.82% resolution by TD, with a face-to-face referral of 49.01% [34], which is consistent with the findings of this study.

This is important to highlight since this evidence suggests that TD is an effective tool in managing at least one-half of the dermatological consultations in primary care, without the need for face-to-face evaluation by the specialist.

As for pharmacological interventions, this study shows a low prescription of drugs. It is important to highlight that a considerable number of skin diseases, especially in the primary healthcare context, can be self-limited or can be efficiently managed with adequate non-pharmacological interventions. This is an important factor to consider when developing drug coverage policies.

Moreover, most patients referred to an in-person evaluation did not receive a pharmacological indication. This correlates with the finding that a drug prescription depends on the patient’s destination, where management in primary care is significantly related to a pharmacological intervention (*p* < 0.05).

From the total number of pharmacological indications (*n* = 85), 80% (*n* = 68) were applicable therapeutic alternatives covered by the government. Although the telemedicine program covers the first-line treatment for a limited amount of diagnosed-based prescriptions, the prescription of drugs covered by either the primary healthcare or the telemedicine program is high, which in turn optimizes access to treatment. As seen on the most frequent pathologies diagnosed in the studied teleconsultations, dermatitis and psoriasis, which are covered by the telemedicine program, account for a considerable portion of the diagnostic suggestions, while other pathologies, such as viral warts, which are also frequent to observe in the primary care, are not. Nonetheless, this study shows that most of the prescribed drugs were within the drugs provided by the public system, optimizing the applicability of the established therapy, as out-of-pocket expenditure or other related factors would not hinder access to treatment. This phenomenon is observed in 100% of the patients who received their treatment in primary care. In contrast, a lower percentage of patients referred to face-to-face care received a drug included in the public plan. Given the aforementioned reasons, we believe that the prescription of drugs covered by the public sector should be a factor of specific evaluation when auditing TD systems. This would promote the use of these pharmaceutical options, educate the consulting dermatologists about these alternatives, or in turn, help develop policies of coverage based on patients’ necessities.

**Figure 3:**
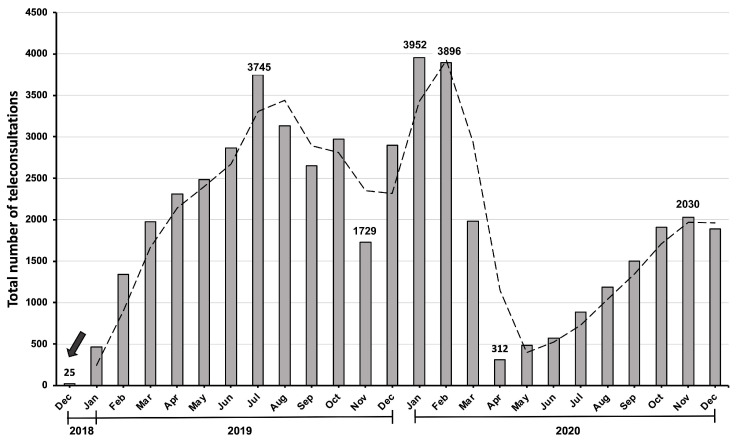
Monthly temporal progression of the total number of teleconsultations to the specialty since the implementation of a national teledermatology platform (marked with arrow) in December 2018 [13]. Adapted from Ref. [13].

Lastly, it is worth mentioning that this study has limitations. First, the study was conducted during the COVID-19 pandemic. For this reason, the consultations received and the patient’s destination after the consultation might have been biased secondary to the epidemiological context at the time. Given the restrictive measures imposed by the Chilean government in 2020 [35], patients’ mobility to either a primary healthcare center or a face-to-face evaluation might have been limited. Knowing that the entrance to the Chilean TD system is via primary healthcare through an in-person consultation with a general physician, the pandemic could have negatively impacted the volume of consults uploaded to the platform. As shown in Figure 3, there is a notable reduction in the teleconsultations evaluated during 2020 as compared to 2019. This is consistent with the overall reduction in consultations to the specialty reported in other settings [36,37] and with the documented reduction of more than 50% of consultations to the specialty observed in the public system in Chile during 2020 [38].

Moreover, there are differences in the use of the TD platform by the different primary healthcare centers across the territory. Some centers use TD as diagnostic support in common dermatoses, while others use it as a triage and referral tool for malignant skin pathology. These differences in the approach of every center to the use of TD as a tool for a specialist evaluation, and to support clinical decision-making could account for notable differences observed between diagnosis, degree of resolution in the primary healthcare, and the number of referrals to secondary care [39].

Nevertheless, the most frequent diagnosis observed and the frequency of referral for face-to-face evaluation showed similar results as compared to larger studies performed in Chile and other countries. In addition, the large sample size of 20,716 electronic consultations and the random extraction of a representative sample size attenuates the effect that the epidemiological situation of the country or the specific use of the platform by individual centers might have played in the consultations.

Finally, it is worth mentioning that strengthening primary care by promoting the educational functions of TD systems is a valuable outcome that promotes network collaboration among the different levels of healthcare attention. This ultimately improves the use of the healthcare system as a unit centered on the user. For these reasons, specific evaluation of the compliance with the educational specifier in TD is an important policy to develop once the most basic specifiers such as diagnostic and therapeutic suggestions are completely fulfilled. In addition, targeted evaluation of the applicability of the response is key to ensuring the proper functioning of the TD systems, as accessible prescriptions ensure the delivery of effective and timely treatment.

## 5. Conclusions

This is the first article that aims to evaluate TD systems through the auditing of data from the Chilean public TD platform since its implementation in December 2018. Aspects such as education, pharmacological indications, and applicability should be the focus of attention to improve TD systems and should be actively evaluated on the audits. On the other hand, the items of diagnosis, diagnostic suggestion, and therapeutic suggestion are present in the majority of the consultations, showing that these systems fulfill their basic functions. In addition, although this study reports a low prescription of pharmacological interventions, the majority of the drugs prescribed were covered either by the telemedicine program or by primary care. This is important to highlight, because, although the telemedicine program covers treatment for a limited number of dermatological diagnoses, when complemented with the drugs available in primary healthcare, most of the patients with a pharmacological indication received the drug at no out-of-pocket expenditure, optimizing prompt and efficient access to treatment. We believe that this experience can help other telemedicine systems implement similar data auditions and serve as feedback tools to improve care systems and evaluate their quality.

## Figures and Tables

**Table 1:** Specifiers evaluated in the response of the teleconsultation.

Specifier	Description
Diagnostic suggestion	The teleconsultation response form is reviewed; the written diagnosis must be included in addition to the ICD 10 coded diagnosis.
ICD 10 Diagnosis	Indicate which final ICD 10 diagnoses were recorded by the dermatologist in the teleconsultation.
Therapeutic suggestion	The teleconsultation form is reviewed; the therapeutic orientations must be clearly recorded. They can be pharmacological or non-pharmacological indications. Pharmacological measures are specified in the following point. Non-pharmacological measures are the general measures provided to each patient for therapy management.
Pharmacological indication	Consider in the pharmacological aspect: medication, dosage, vehicle, and time of treatment.
Therapeutic applicability	This indicator applies if the case is subject to pharmacological indication. The therapeutic suggestion must be in accordance with what is included in the basket of drugs provided by the public healthcare system.
Education	The therapeutic suggestion must provide educational content to the primary healthcare physician: Practical advice for case management.
Destination of the patient after the consultation	The teleconsultation form must have marked in the indications tab: Option 1: Management at the primary healthcare center Option 2: Secondary Face-to-face referral Option 3: others: Referral to the medical ward, referral to another specialty (indicating which one), or incomplete data. Assessment of the number of patients that continue in the primary healthcare center via teledermatology is evaluated at this point.

**Table 2:** Guideline for compliance with the specifiers evaluated in the response to the teleconsultation.

Aim of the Indicator	Indicator	Error Margin	Compliance Threshold	Verifiers	Checklist
To Evaluate compliance in completing the basic content required for the response to the teleconsultation.	Number of asynchronous tele-consultations that comply with the minimum contents required for the response to the teleconsultation in a period of 1 year	5%	≥80%	Representative Sample size calculation out of the universe of teleconsultations performed in 2020, provided by the Superintendencia de Salud. Randomly selected cases. The response must include: 1. Diagnostic suggestion 2. ICD 10 diagnosis 3. Therapeutic suggestion 4. Therapeutic indication 5. Applicability of the response. 6. Education. 7. Destination of the patient after the consultation.	Complies/does not comply

## Data Availability

The data presented in this study are available on request from the corresponding author.
